# Size-Tunable Magnetite Nanoparticles from Well-Defined Iron Oleate Precursors

**DOI:** 10.1021/acs.chemmater.2c02046

**Published:** 2022-08-16

**Authors:** Kyle M. Kirkpatrick, Benjamin H. Zhou, Philip C. Bunting, Jeffrey D. Rinehart

**Affiliations:** ^†^Department of Chemistry and Biochemistry and ^‡^Materials Science and Engineering Program, University of California, San Diego, La Jolla, California 92093, United States

## Abstract

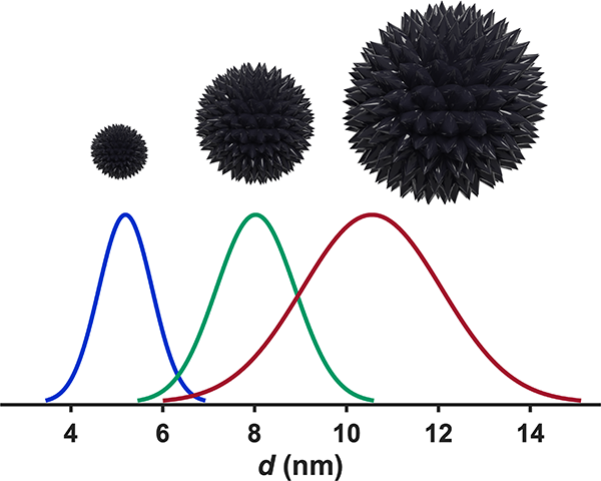

The synthesis of iron oxide nanoparticles with control over size and shape has long been an area of research, with iron oleate being arguably the most successful precursor. Issues with reproducibility and versatility in iron oleate-based syntheses remain, however, in large part due to the mutable nature of its structure and stoichiometry. In this work, we characterize two new forms of iron oleate precursor that can be isolated in large quantities, show long-term stability, and have well-defined stoichiometry, leading to reproducible and predictable reactivity. Synthesis with these precursors is shown to produce iron oxide nanoparticles in a tunable size range of 4–16 nm with low size dispersity and properties consistent with magnetite in the superparamagnetic size regime.

## Introduction

The thermal decomposition of metal coordination complexes is a long-standing and commonly employed technique for producing colloidal nanoparticles.^1−4^ Complexes formed from transition metals and the oleate anion [Ol^–^ = CH_3_(CH_2_)_7_CH=CH(CH_2_)_7_COO^–^] are some of the most common precursors for nanoparticles, providing access to a wide variety of metal oxide (e.g., CoO,^5,6^ MnO,^7^ and ZnO^8^) and metal ferrite [e.g., MFe_2_O_4_ (M = Fe, Co, Ni, Mn, or Zn)]^9−11^ materials. Perhaps the greatest testament to the utility of the oleate precursor is its continued dominance, despite persistent issues in reproducibility and predictability. Much of this variability arises from the fact that the term “metal oleate” rarely refers to an exact molecular formula. Indeed, there is significant evidence that metal oleates are highly variable materials for which the ligation, nuclearity, solvation, and oxidation state are sensitive to a host of synthetic details.^12,13^ This sensitivity presents a challenge to sample-to-sample consistency, inhibiting predictive and scalable control over nanoparticle size, size dispersity, morphology, and phase. Often, even small alterations to a functioning synthesis require extensive re-optimization. Once optimized, however, control over nanoparticle phase, size, and shape has been demonstrated to impressive levels of precision.^13−16^

While the issues arising from precursor variability are a common challenge among nanoparticle syntheses, reliable methods are especially vital for magnetic nanomaterials. Even slight variations in phase, morphology, homogeneity, and heterostructure can limit or even negate their functional magnetic capabilities.^17^ Especially with iron oxide, these capabilities are vital for optimizing responses in applications such as magnetic hyperthermia,^11,18^ nanocomposite magnetoresistance,^19−23^ smart fluids,^24^ magnetic particle imaging,^25−27^ magnetic particle spectroscopy,^28,29^ and thermometry.^30,31^

Many syntheses for iron oxide nanoparticles have been explored from a host of precursors, including long chain carboxylates (oleate,^2,12^ stearate,^32−34^ and palmitate^16^), acetate,^35,36^ acetylacetonate,^1,37^ carbonyl,^38^ carbonate,^39^ and hydroxide ligands.^40,41^ Among the many demonstrated precursor materials, iron oleate is arguably the most popular, as it is nontoxic, can be made on a large scale,^2^ and has been shown to produce particles of a variety of sizes (*d* = 1–40 nm),^13,14^ with considerable shape control.^15,16^

Many research groups,^10,12,14,42^ including our own,^23^ have reported structural and magnetic data for magnetite nanoparticles using iron oleate syntheses, yet somewhat counterintuitively, these syntheses continue to have challenges. The formulations of the iron oleate used as a synthetic precursor to magnetic nanoparticles have been shown, through careful characterization, to be highly sensitive to minor variations in its synthesis due to its propensity to retain water, oleic acid, and other reaction byproducts.^12,43,44^

**Scheme 1:**
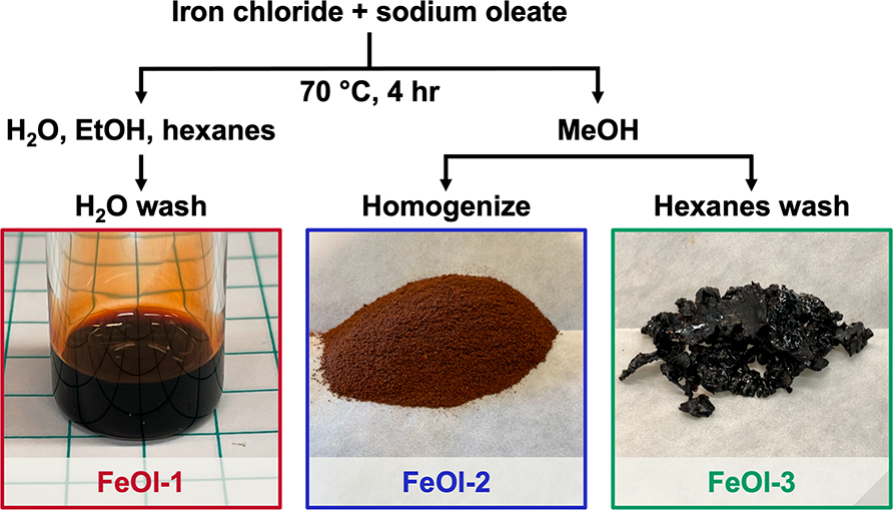
Abbreviated Reaction Scheme and Representative Images of Iron Oleate Precursors

Herein, we compare our standard preparation of the viscous red-brown oil typically characterized as iron oleate (FeOl-1) to two newly isolated iron oleate starting materials: a fine dark brown powder preparation (FeOl-2) and a hard, waxy preparation (FeOl-3) (Scheme 1). Similar to FeOl-1, FeOl-2 and -3 lack crystallographic order yet are compositionally more consistent and, upon thermal decomposition, lead to low-size dispersity nanoparticles in a systematic and tunable size range of 4–16 nm. In this work, we detail the synthetic methods and characterization of these new precursors for the thermal decomposition synthesis of magnetite nanoparticles. Additionally, we discuss the possible advantages of using FeOl-2 and -3 in nanoparticle synthesis in the context of both practical synthetic methods and the resulting magnetism. A general reaction scheme for the synthesis of each iron oleate precursor described in this work is depicted in Scheme 1.

## Experimental Section

### Materials

The reagents used were iron(III) chloride hexahydrate (97%, Alfa Aesar), iron(II) chloride tetrahydrate (97%, Fisher), sodium oleate (97%, TCI), oleic acid (90%, Alfa Aesar), and 1-octadecene (90%, Sigma-Aldrich). ACS grade hexane, ethanol, and methanol were purchased from Fisher. Oleic acid was degassed and stored under vacuum in a Schlenk flask covered with aluminum foil. All other chemicals were used as received.

### Safety Considerations for Pressure Flasks

The use of a pressure flask represents a convenient and green alternative to flowing water reflux condensers. Safety is paramount when heating closed vessels. The Ace pressure flasks are rated for 60 psig at 120 °C, which is well above the calculated pressure of this reaction performed at 70 °C.

### Synthesis of FeOl-1

In a 250 mL Ace round-bottom pressure flask with a thermowell (rated for 60 psig at 120 °C), iron(III) chloride hexahydrate (4.05 g, 15 mmol) and sodium oleate (13.70 g, 45 mmol) were mixed with deionized (DI) water (30 mL), ethanol (23 mL), and hexanes (53 mL). The flask was sealed and heated to 70 °C for 4 h. After the flask had been cooled to room temperature, the upper organic layer of the reaction was separated and washed with DI water (∼50 mL) in a separatory funnel. The hexane was removed via vacuum, resulting in a viscous, dark red solid that was further dried in a vacuum oven (70 °C, house vacuum) for 24 h.

### Synthesis of FeOl-2

In a 250 mL Ace round-bottom pressure flask with a thermowell (rated for 60 psig at 120 °C), iron(III) chloride hexahydrate (4.05 g, 15 mmol) and sodium oleate (13.70 g, 45 mmol) were mixed with methanol (105 mL). The flask was sealed and heated to 70 °C for 4 h. After the flask had been cooled to room temperature, a dark yellow clump was collected and washed with DI water (100 mL), forming a brown rubbery solid. The brown rubbery solid and 250 mL of deionized water were added to a 250 mL Erlenmeyer flask. A tissue homogenizer (IKA Works T25 Basic S1) was used to simultaneously break up and wash the rubbery solid, converting it to a fine powder and removing residual sodium chloride and sodium oleate. The homogenization process was carried out for 0.5 h, followed by a vacuum filtration to recover the powder. An additional homogenization step was performed with 250 mL of DI water for 0.5 h. The powder was collected again and dried in a vacuum oven (70 °C, house vacuum) for 24 h before being used in nanoparticle syntheses.

### Synthesis of FeOl-3

In a 250 mL Ace round-bottom pressure flask with a thermowell (rated for 60 psig at 120 °C), iron(III) chloride hexahydrate (2.70 g, 10 mmol), iron(II) chloride hexahydrate (1.00 g, 5 mmol), and sodium oleate (13.70 g, 45 mmol) were mixed with methanol (105 mL). The flask was sealed and heated in a mantle using a PID controller to 70 °C for 4 h. After the flask had been cooled to room temperature, the viscous brown product was dissolved in 40 mL of hexanes and washed with DI water (100 mL) in a separatory funnel. The iron oleate was left in hexanes. One milliliter of the hexane solution was dried and weighed to determine the total amount of iron oleate. 1-Octadecene was added to the iron oleate solution to make a 1:1 (w/w) stock solution. The hexane was removed via vacuum, and the stock solution was dried in a vacuum oven (70 °C, house vacuum) for 24 h before being used in nanoparticle syntheses.

### Nanoparticle Synthesis from FeOl-2

Fe_3_O_4_ nanoparticles were synthesized according to modified literature procedures.^2^ In a typical synthesis, FeOl-2 was mixed with oleic acid (Table S1) in a 50 mL three-neck Morton flask and placed in a vacuum oven (70 °C, house vacuum) for 1 h. During this step, a stir bar was added and used to mix the FeOl-2 and OA, ensuring a homogeneous product. This step helps to react the FeOl-2 and oleic acid, preventing FeOl-2 from being deposited on the upper half of the flask during the degas and heat up. Without this step, it is difficult to avoid accumulation of the unreacted solid FeOl-2 on the sides of the flask during degas and heating, which can affect the reaction outcome. Afterward, 1-octadecene was added according to Table S1. The flask was equipped with a temperature probe (left neck), condenser (middle neck), and flow adapter (right neck) and then placed in a heating mantle. The reaction mixture was degassed and backfilled with dinitrogen three times at room temperature. The reaction mixture was heated to 110 °C and degassed under vacuum for 0.5 h, after which the atmosphere was backfilled with dinitrogen. Throughout the reaction, dinitrogen (100 sccm) was passed through a side neck of the Morton flask and out the top of the condenser, attached to an oil bubbler. The reaction mixture was heated to reflux at a rate of 3.3 °C/min using a PID controller and refluxed for 0.5 h. When the temperature reached reflux, dioxygen (5 sccm) was added to the dinitrogen stream and flowed until the end of the reaction.

The reflux temperature was recorded, and the timer was started when vigorous bubbling began. The stir rate of the reaction was kept to a minimum (500 rpm) during heat-up and increased (1100 rpm) at 300 °C. This is necessary to keep material within the reaction mixture during heat-up.

### Nanoparticle Synthesis from FeOl-3

Fe_3_O_4_ nanoparticles were synthesized according to modified literature procedures.^2^ In a typical synthesis from the iron oleate stock solution, 2.00 g of the stock solution (1.00 g of FeOl-3 in 1.00 g of 1-octadecene) was mixed with oleic acid (0.20 g) and additional 1-octadecene (6.00 g) in a 50 mL three-neck Morton flask. The flask was equipped with a temperature probe (left neck), condenser (middle neck), and flow adapter (right neck) and then placed in a heating mantle. The reaction mixture was degassed and backfilled with dinitrogen three times at room temperature. The reaction mixture was heated to 110 °C and degassed under vacuum for 0.5 h, after which the atmosphere was backfilled with dinitrogen. Throughout the reaction, dinitrogen (100 sccm) was passed through a side neck of the Morton flask and out the top of the condenser, attached to an oil bubbler. The reaction mixture was heated to reflux at a rate of 3.3 °C/min using a PID controller and refluxed for 0.5 h. When the temperature reached reflux, dioxygen (5 sccm) was added to the dinitrogen stream and flowed until the end of the reaction.

### Purification of Fe_3_O_4_ Nanoparticles

Nanoparticles were isolated and purified by the addition of hexanes and ethanol in a 1:1 ratio, followed by centrifugation (7 min at 8500 rpm). The nanoparticles were redispersed in hexanes. Two more cycles of purification by precipitation with ethanol and centrifugation were carried out before the nanoparticles were stored in hexanes.

### Characterization

Transmission electron microscopy was carried out using a FEI Spirit transmission electron microscope (TEM) operating at 120 kV, with images collected by a 2K × 2K Gatan CCD camera. TEM samples were prepared by drop-casting and air drying a dilute solution of nanoparticles in hexanes onto a carbon-coated copper TEM grid. Particles were analyzed in ImageJ using the default thresholding algorithm of sample sizes exceeding 500 particles for all syntheses.

IR measurements were carried out using a Bruker Alpha FT-IR spectrometer.

MALDI-MS was carried out on a Bruker Autoflex Max. Bruker peptide calibration standard II (bradykinin fragments 1–7, angiotensin II, angiotensin I, Substance P, Bombesin, Renin Substrate, ACTH clip 1–17, ACTH clip 18–39, and somatostatin 28) in a HCCA matrix was used as a calibrant. Samples were mixed with a 9-nitroanthracene matrix in chloroform. A pulsed nitrogen laser (337 nm) with a power setting of 55% was used with a 21 kV potential operating in positive ion mode.

Magnetic measurements were carried out using a Quantum Design MPMS3 SQUID magnetometer. Nanoparticle samples were dried to a fine powder (1–2 mg), loaded into a VSM sample holder, and secured in a plastic straw.

The iron concentration was determined by ICP-MS. Samples were digested in 70% HNO_3_ (trace metal grade), diluted to 3% HNO_3_ with milli-q water, and analyzed by a Thermo iCAP RQ ICP-MS instrument. Elemental analysis was performed on a PerkinElmer PE2400-Series II, CHNS/O analyzer. Powder X-ray diffraction was performed with a Bruker D8 Advance diffractometer using Cu Kα (1.5418 Å) radiation (40 kV, 40 mA) or a Bruker Apex II Ultra CCD instrument using Mo Kα (λ = 0.71073 Å) radiation.

## Results and Discussion

### Synthesis and Characterization of FeOl-1

The oleate precursor for the synthesis of iron oxide nanoparticles is generally agreed to contain the ubiquitous trinuclear oxo-centered iron motif, [Fe_3_O]^*n*+^, with charge balance provided by chelating oleate ligation.^10^ Because there are countless variations on iron oleate syntheses, we use the general name FeOl-1 as a means to delineate observations on the basis of procedures and characterization described herein for comparison with the materials FeOl-2 and FeOl-3.

Synthesis for FeOl-1 proceeds by stirring a solution of iron(III) chloride and sodium oleate in DI water, ethanol, and hexanes at 70 °C for 4 h (Scheme 1).^2^ Afterward, the hexanes phase containing FeOl-1 is washed with DI water using a separatory funnel. The hexanes are removed under reduced pressure until the highly viscous, dark red oil (FeOl-1) is obtained. Literature procedures for this synthesis vary greatly and offer insight into how the starting material formulation can affect the resultant nanoparticle properties.

Unlike a well-defined, crystalline molecular structure, the connectivity and composition of FeOl-1 can change dramatically during synthesis and workup as a result of reflux temperature, reaction solvent, volume of solvent, degree of diligence in byproduct extraction, and method of solvent removal. For instance, the reported reflux temperature from syntheses similar to that of FeOl-1 varies from 57 to 70 °C,^2,14^ with this variability exhibiting important consequences on the resultant nanoparticles. Bronstein et al. have also demonstrated the partial removal of free/residual oleic acid (OA) from syntheses similar to that of FeOl-1, indicating a high potential for variability in stoichiometry.^12^

**Figure 1:**
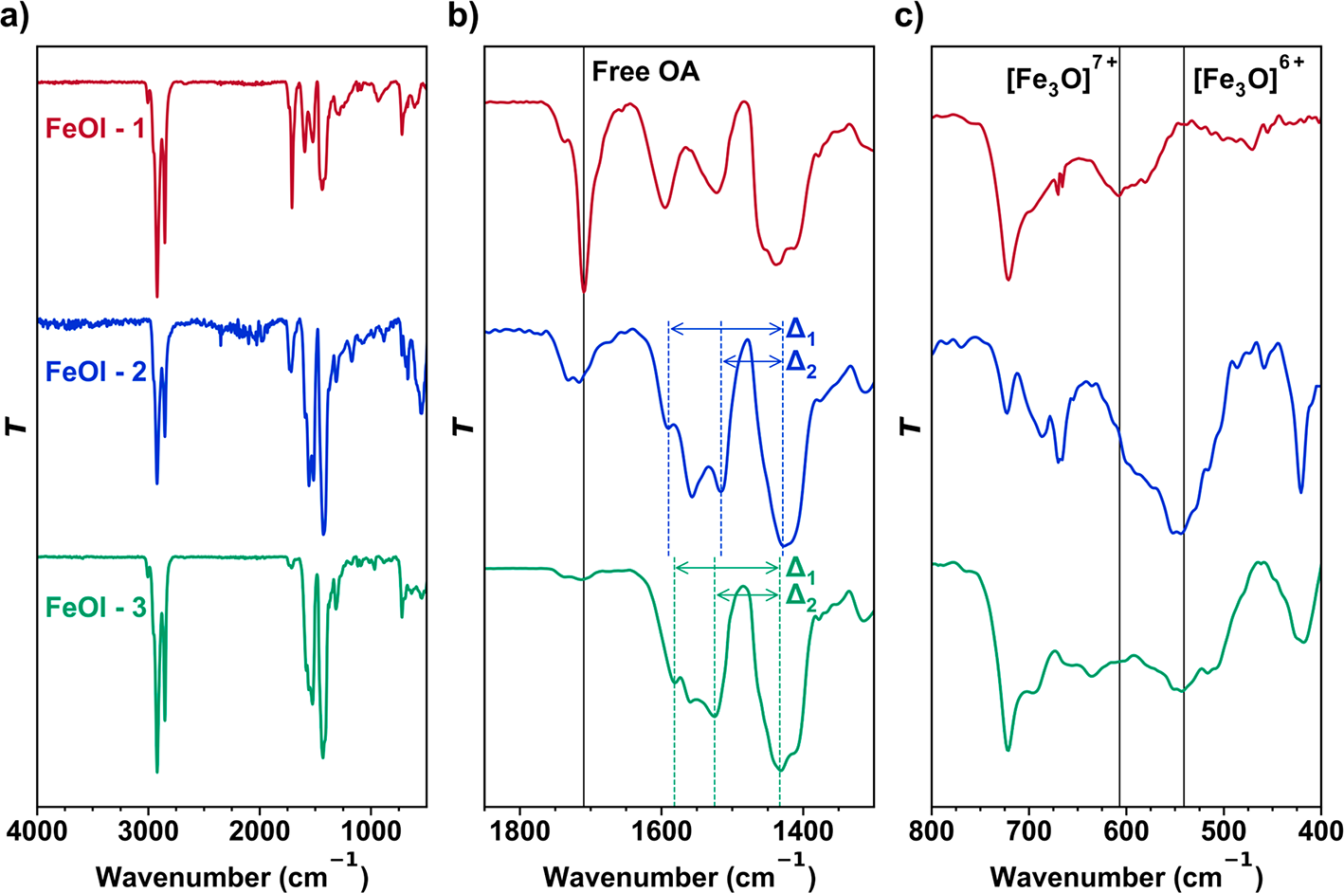
Infrared characterization of iron oleate precursors plotted as wavenumber vs normalized transmittance. (a) Full infrared spectra. (b) Carboxylate region with free/residual oleic acid (OA) indicated at 1710 cm^–1^. (c) Metal–oxo core region with [Fe_3_O]^*n*+^ cluster peaks indicated at 610 cm^–1^ (*n* = 7) and 545 cm^–1^ (*n* = 6).

Additionally, the charge of the trinuclear iron–oxo cluster leads to further inconsistency. The mixed valence cluster [Fe_3_O]^6+^ leads to a net neutral molecule with six oleate anions, yet X-ray photoelectron spectroscopy (XPS) on material from syntheses similar to that of FeOl-1 is consistent with an all-Fe^3+^ core.^12^ To further support this, the net charge of the iron–oxo core can be probed by peaks in the region of 500–650 cm^–1^ of an infrared (IR) spectrum.^10^ Analysis of molecular iron–oxo clusters indicates that an all-Fe^3+^ core exhibiting *D*_3*h*_ symmetry will show a peak at ∼610 cm^–1^, which we indeed observe in FeOl-1 (Figure 1c). Therefore, the [Fe_3_O]^7+^ cluster core predicted for FeOl-1 has an uncompensated cationic charge, most likely resulting in anionic outer sphere oleate.

In addition to residual anionic oleate present in FeOl-1, residual OA is also incorporated due to the nature of the biphasic synthesis, evident by the peak at ∼1710 cm^–1^ in its IR spectrum (Figure 1b). Combined with residual OA present in the material, even in a purified form, FeOl-1 contains minimum excess ligand of roughly 30% by mass. Upon determination of the carbon and hydrogen percentages through elemental analysis (EA) and iron percentage through inductively coupled plasma mass spectrometry (ICP-MS), FeOl-1 matches the formula of [Fe_3_O(oleate)_6_][oleate]·(oleic acid)_2_·H_2_O (Table 1), as noted by others.^12,13^ This composition, however, has been shown to vary as a function of storage, reaction, and workup conditions. Additionally, some syntheses use stoichiometries based on an assumed formula of Fe_3_O(oleate)_6_ or Fe(oleate)_3_, leading to higher uncertainty in the metal:ligand ratios. Typical nanoparticle syntheses add OA as a surfactant, and because of the viscous and cationic nature of the core cluster in FeOl-1, it is very difficult to fully separate from free OA, oleate anions, residual sodium, and other solvents. Thus, reproducing metal:surfactant ratios across different batches of starting material and in different laboratories is a recurring challenge.

**Table 1:** Elemental Composition of Iron Oleate Precursors with Corresponding Formulas^a^

	C	H	Fe
FeOl-1	71.19 ± 0.08	10.50 ± 0.31	6.51 ± 0.72
[Fe_3_O(oleate)_6_][oleate]·(oleic acid)_2_·H_2_O	71.10	11.09	6.12
FeOl-2	62.34 ± 0.01	9.22 ± 0.02	15.27 ± 1.20
Fe_3_O C_55_H_103_O_7_	62.32	9.8	15.81
FeOl-3	67.42 ± 0.11	10.31 ± 0.01	9.40 ± 0.62
Fe_3_O(oleate)_6_·3H_2_O	67.34	10.67	8.70

a Evidence and further explanation of the formula composition are given in the text.

The drying step contains two other seemingly innocuous variables that lead to reproducibility issues. Multiple studies have analyzed the impact of drying time and temperature on the metal–carboxylate binding mode distribution from syntheses similar to that of FeOl-1.^12,18,43,44^ Balakrishnan et al. observed a diminishing signal from free OA with increased drying times, attributing it in part to the removal of crystal hydrate water.^44^ This change in the binding mode distribution led to a dramatic change in the resulting nanoparticles, from 6 to 13 nm for drying times from 5 to 30 days, respectively.^44^ Although the drying step represents a convenient parameter for tuning the size in this case, a precursor that changes its composition over time is not ideal.

### Iron Oleate Characterization of FeOl-2

Initial attempts to obtain a simplified formulation of FeOl-1 resulted in promising results from syntheses proceeding in organic solvents, specifically in methanol (MeOH). An optimized procedure was developed wherein FeOl-2 was prepared in a sealed Ace pressure vessel by stirring iron(III) chloride and sodium oleate in MeOH at 70 °C, followed by washing with DI water, homogenization of the resulting solid, and drying. A detailed step-by-step demonstration for the synthesis of FeOl-2 with photos is shown in Figure S1. Via this procedure, FeOl-2 is isolated as an air-stable powder in gram scale quantities.

Homogenization in aqueous suspension was used to break up the tough rubbery clumps formed upon initially isolating the reaction mixture from MeOH (Scheme 1). The thorough aqueous homogenization was found to be crucial for the removal of residual sodium chloride and sodium oleate, as confirmed by powder X-ray diffraction analysis (Figure S2). Following collection of the solid via vacuum filtration and drying (70 °C, 24 h, house vacuum), FeOl-2 was isolated as a fine, dark brown powder (Scheme 1) and used in nanoparticle syntheses without further purification. Importantly, FeOl-2 is found to be a convenient nanoparticle starting material, as it can be synthesized with consistent stoichiometry and readily converts to Fe_3_O(oleate)_6_ in the presence of excess OA (e.g., in precursor solutions for magnetite nanoparticles).

With FeOl-1 leading to a viscous oil and FeOl-2 leading to an insoluble powder, we sought methods for pinpointing key differences in composition, connectivity, and/or oxidation state. As in FeOl-1, IR spectroscopy was used to probe the metal–carboxylate binding modes. The IR spectrum of FeOl-2 was found to be similar to that of FeOl-1, with metal–carboxylate peaks corresponding to symmetric and asymmetric stretching modes presenting in the region between 1300 and 1800 cm^–1^ (Figure 1).^12^ Four binding configurations are possible: ionic, monodentate, bridging, and bidentate. The latter two are most commonly observed. The most probable binding mode can be predicted by the difference (Δ) between the symmetric and asymmetric peaks, with Δ = 140–200 cm^–1^ corresponding to a bridging mode and Δ < 110 cm^–1^ corresponding to a bidentate mode.^12^ Via this analysis, FeOl-2 exhibits bridging (1592 cm^–1^; Δ_1_ = 163 cm^–1^) and bidentate modes (1514 cm^–1^; Δ_2_ = 85 cm^–1^), varying significantly from the IR spectrum of FeOl-1. Bronstein et al. observed a similar IR spectrum after washing with acetone and ethanol, attributing the change in Δ to a more regular packing of the oleate ligands following the removal of free OA.^12^ Notably, FeOl-2 lacks a free OA carbonyl peak at 1710 cm^–1^ when fully purified. This spectroscopic signature can be used to prevent stoichiometric errors due to variable free OA, which can be difficult to remove from FeOl-1.

While FeOl-1 exhibits a strong peak at ∼610 cm^–1^ corresponding to a [Fe_3_O]^7+^ core, FeOl-2 exhibits a shifted peak at ∼550 cm^–1^, suggesting a localized mixed valence [Fe_3_O]^6+^ core.^10^ Although not definitive evidence of the valence state, this shift is consistent with the local symmetry decreasing from (pseudo) *D*_3*h*_ to *C*_2*v*_ expected for reduction at a single metal center.

Given the possibility of a partially reduced metal cluster core, we were interested in exploring whether FeOl-2 formed via a more complex reactivity than expected. The [Fe_3_O]^6+^ motif has been shown to be catalytically active and convert olefinic alcohol acetates into epoxides, likely forming aldehydes in the process.^45,46^ To probe the importance of the reactivity of olefins in the presence of [Fe_3_O]^6+^, the elemental composition of FeOl-2 was analyzed by EA and ICP-MS (Table 1). The Fe:C ratio for FeOl-2 (1:18) was well below that expected for Fe_3_O(oleate)_6_ (1:36), consistent with an irreversible loss of oleate or a breakdown of oleate into a smaller carboxylate. The redox activity of the iron–oxo cluster is consistent with some mechanisms for this oleate reactivity. Interestingly, such reactivity could be very difficult to characterize for in situ preparations or preparations where significant excess oleate is present and thus could contribute to general reproducibility issues in many oleate-based precursors.^39^

To further probe the reactivity of the [Fe_3_O]^6+^ cluster, we used headspace gas chromatography-mass spectrometry (GC-MS) experiments to monitor for any gaseous byproducts (e.g., aldehydes) released during the synthesis of iron oxide nanoparticles. FeOl-2 was reacted with OA in a GC vial and heated to 70 °C, simulating the degas step prior to a typical nanoparticle reaction. The vial headspace was sampled for 10 min at 70 °C and corrected for a background of neat OA. The chromatograph (Figure S3) shows the formation of aldehydes in sequential sizes, ranging from pentanal to nonanal. An identical experiment was performed with FeOl-1 in OA. Again, aldehydes ranging from C_5_ to C_9_ are observed (Figure S4). Cleavage of the alkene in OA likely proceeds through an epoxidation step at the trinuclear iron–oxo cluster, followed by addition of water, forming a diol.^47,48^ Finally, the diol can be oxidized to an aldehyde by ambient oxygen. The GC-MS experiments demonstrate the redox activity of iron oleate-based precursors in ligand decomposition reactions. These results bolster synthetic methods that include complete removal of water and oxygen from the system at low temperatures to minimize reactivity from uncontrolled side reactions catalyzed by metal–oxo cluster reagents.

To test the batch-to-batch reproducibility, the synthesis of FeOl-2 was performed in triplicate using the standardized procedure. The metal carboxylate behavior and elemental composition of the three batches were analyzed via IR and ICP/EA, respectively. The peaks in the IR spectra are functionally identical (Figure S5), and the elemental composition remains quite similar (Table S1), with RSD (%) values of 1.7, 0.50, and 1.6 for the percentages of Fe, C, and H, respectively.

Although noncrystalline, FeOl-2 is a free-flowing powder that can be made reproducibly with well-defined molar ratios and is thus amenable to use as a starting material. From a synthetic standpoint, a powder is simple to manipulate. FeOl-2 is air-stable and can be made in large quantities, enabling a potential scale-up of the nanoparticle reaction. After being stored in air for six months, FeOl-2 exhibits a nearly identical IR spectrum (Figure S6), demonstrating a high degree of air stability; however, considering the reactivity of the [Fe_3_O]^6+^ cluster, storage of FeOl-2 under an inert atmosphere is preferred.

**Figure 2:**
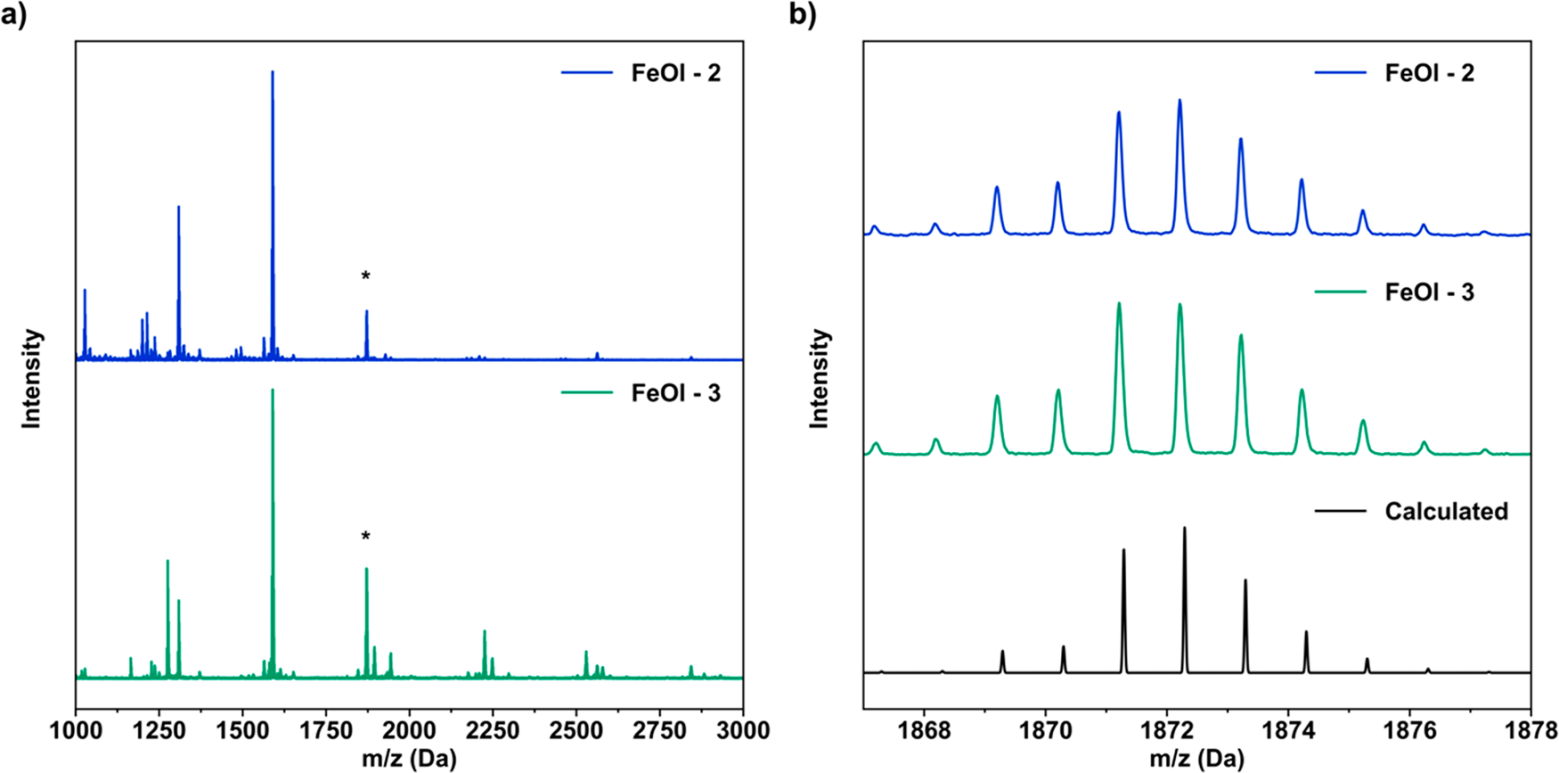
Molecular cluster structural data from MALDI-MS plotted as intensity vs *m*/*z*. (a) Full MALDI-MS spectra of FeOl-2 and -3. The asterisk indicates the *m*/*z* value of the [Fe_3_O(oleate)_6_]^+^ ion. (b) Magnified view of the asterisk-marked molecular ions of FeOl-2 and -3 compared to the calculated isotope pattern of [Fe_3_O(oleate)_6_]^+^.

Finally, because of its extended solid properties, FeOl-2 is highly insoluble in common organic solvents. However, it reacts with OA and mild heat (70 °C), allowing for dissolution in hexanes. This permits characterization by matrix-assisted laser desorption ionization mass spectrometry (MALDI-MS) for a detailed investigation of its cluster size and molecular weight with minimal fragmentation. The MALDI-MS spectrum of FeOl-2 in OA (Figure 2a) exhibits peaks corresponding to the expected trinuclear iron–oxo cluster, Fe_3_O(oleate)_6_. The molecular cluster [Fe_3_O(oleate)_6_]^+^ is observed at 1872 Da (Figure 2b). Further confirming the assignment, the fragments [Fe_3_O(oleate)_5_]^+^ and [Fe_3_O(oleate)_4_]^+^ at 1570 and 1320 Da, respectively, are observed due to the sequential loss of oleate ligands.

These data are consistent with the idea that, following introduction of OA and mild heating, FeOl-2 is converted in situ to a form similar to that of FeOl-1 in a nanoparticle synthesis. This “activation” process of FeOl-2 extends to other carboxylic acids. For example, heating FeOl-2 with lauric acid in hexanes shows the presence of Fe_3_O(laurate)_6_ by MALDI-MS (Figure S7). Considering the characterization of the material conducted thus far, we propose FeOl-2 to be an extended solid based on [Fe_3_O]^6+^ clusters bound by carboxylates and capable of in situ activation by OA for the synthesis of iron oxide nanoparticles.

### Iron Oleate Characterization of FeOl-3

While storage and preparation were simplified by the insolubility of FeOl-2, extra care is required during the workup procedure to remove impurities. Achieving a soluble Fe_3_O(oleate)_6_ cluster in the initial reaction mixture was likely to simplify purification from byproducts and residual salts. To do this, a procedure identical to that of FeOl-2 was followed with a mixture of iron(III) chloride and iron(II) chloride (2:1). The oily solid product of this reaction could be dissolved in hexanes, making a single aqueous wash usually sufficient for removing impurities. Hexanes were removed under reduced pressure to form a dark brown waxy solid [FeOl-3 (Scheme 1)]. Solid FeOl-3 was found to be suitable for synthesis directly or via formation of an octadecene stock solution for more convenient manipulation. The stock solution can be made by the addition of octadecene to FeOl-3 or directly to the hexane solution isolated after washing with water.

The IR spectrum of FeOl-3 exhibits peaks similar to those of FeOl-2, with a bridging mode at 1580 cm^–1^ (Δ_1_ = 150 cm^–1^) and a bidentate mode at 1526 cm^–1^ (Δ_2_ = 96 cm^–1^). The free OA peak near 1710 cm^–1^ is almost entirely absent. Additionally, IR data suggest the iron–oxo core of FeOl-3 is mixed valence, [Fe_3_O]^6+^, due to the observation of a peak observed at 550 cm^–1^.^10^

In contrast with the insolubility of FeOl-2, FeOl-3 is soluble in hexanes and can be analyzed by MALDI-MS directly to determine the cluster size and molecular weight. The MALDI-MS data indicate that FeOl-3 consists of the trinuclear iron–oxo cluster, Fe_3_O(oleate)_6_, matching the calculated molecular ion at 1872 Da (Figure 2a). The two subsequent fragments at 1570 and 1320 Da are due to the loss of sequential oleates, confirming this assignment (Figure 2b).

Elemental analysis (Table 1) closely matches a solvated formula of Fe_3_O(oleate)_6_·3H_2_O. With these data, the assignment of FeOl-3 to a mixed valence, trinuclear iron–oxo cluster, Fe_3_O(oleate)_6_, with no residual free oleic acid or anionic oleate is corroborated by elemental, IR, and MS analysis.

Both FeOl-2 and FeOl-3 provide practical advantages as starting materials for magnetic nanoparticle synthesis. There are still details of their structure and reactivity to explore, and we continue to do so. Their ultimate utility, however, lies in whether the observed stability and well-defined stoichiometry can be leveraged to enhance control over size and phase purity in iron oxide (especially magnetite) synthesis.

### Iron Oxide Nanoparticle Synthesis

**Figure 3:**
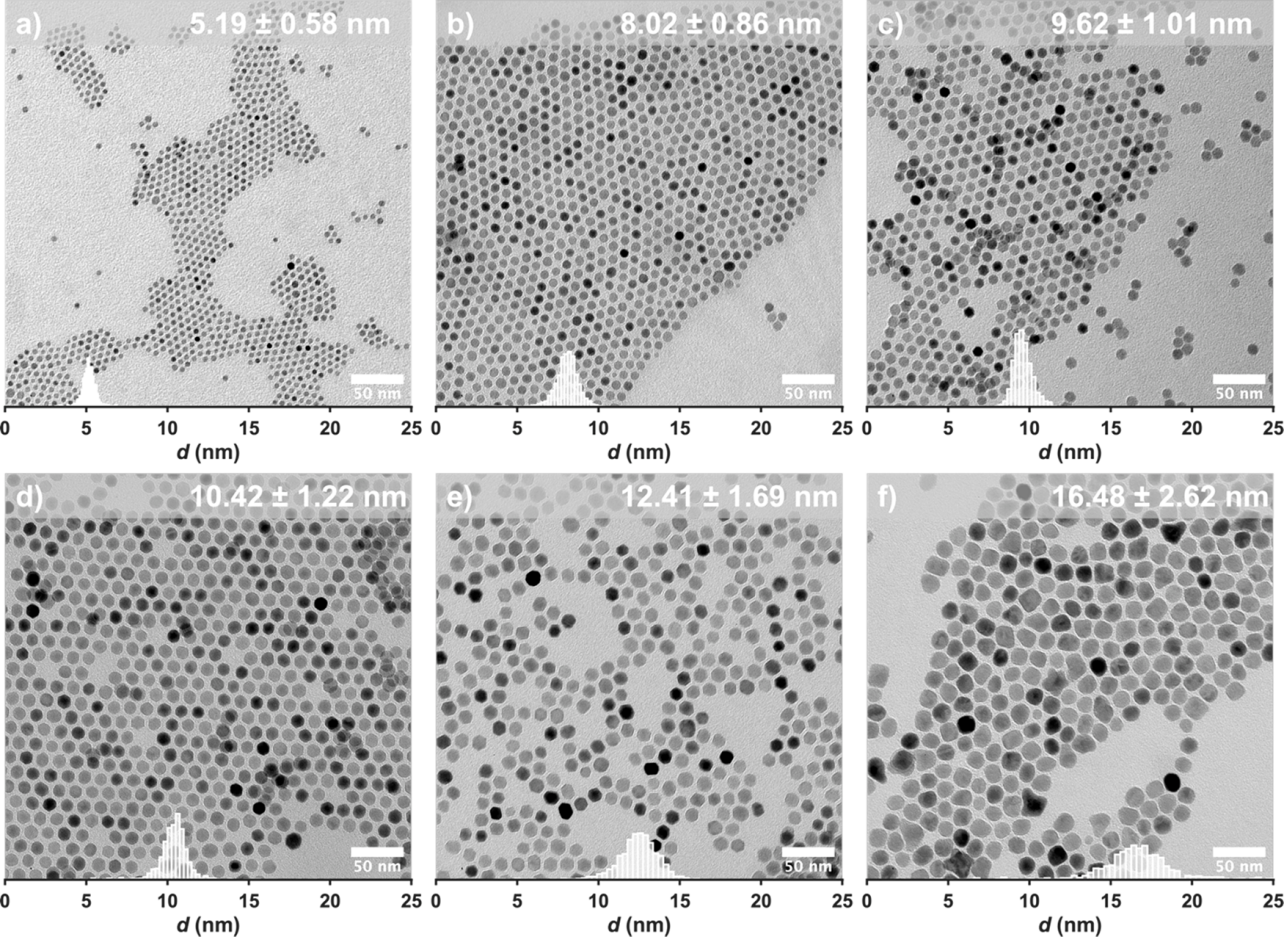
Representative TEM images of nanoparticles synthesized from FeOl-2 with overlaid size histograms.

The synthesis of iron oxide nanoparticles from FeOl-2 is adapted from literature procedures with modifications (Figure S8).^2,23,42^ Briefly, a stirred solution of FeOl-2 was heated under active evacuation of the headspace to 110 °C. After reaching 110 °C, the solution was evacuated for a further 30 min and then heated to reflux under a N_2_ flow (100 sccm). Once reflux had been achieved, a stream of O_2_ (5 sccm) in N_2_ (100 sccm) was passed through the reaction vessel to ensure magnetite phase purity and the reaction was allowed to continue for an additional 30 min. Representative nanoparticle samples in the range of 5–16 nm are shown in Figure 3 and Table 2, along with a full table of synthetic conditions (Table S2). Two tests of reproducibility are shown for 10.5 nm nanoparticles (Figure S9) and 12.5 nm nanoparticles (Figure S10).

**Table 2:** Nanoparticle Size and Size Dispersity under Varying Synthetic Conditions

size (nm)	RSD (%)	OA:Fe	Fe % (w/w)
Nanoparticles from FeOl-2			
5.19	11.2	2.0	0.5
8.02	10.7	1.0	0.5
9.62	10.5	1.0	2.0
10.42	11.7	1.5	1.0
12.41	13.6	1.0	1.0
16.41	15.9	0.5	1.5
Nanoparticles from FeOl-3			
4.24	10.8	1.5	0.9
4.99	12.2	0.7	1.3
5.19	12.9	1.5	1.4

**Figure 4:**
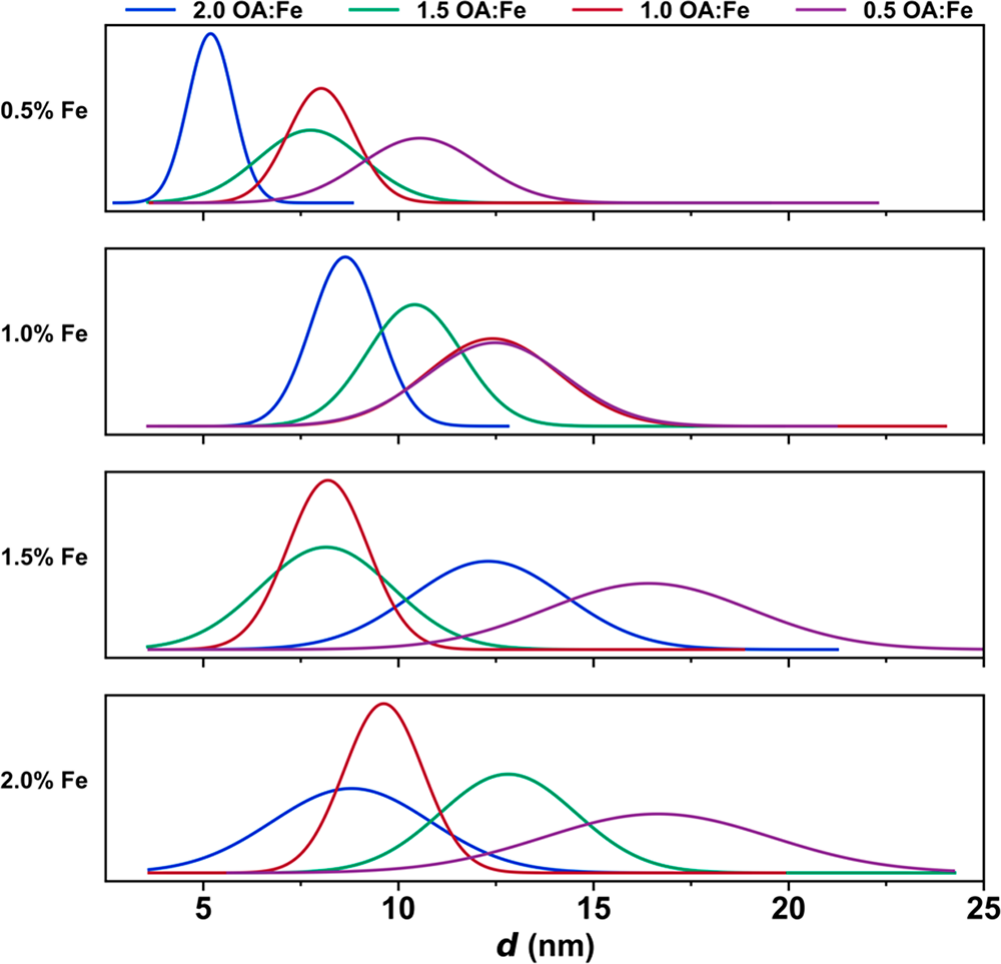
Nanoparticles synthesized from FeOl-2 were fit to normal distributions, demonstrating the effect of varying Fe % (w/w) and OA:Fe on nanoparticle size and size dispersity.

As a result of the convenient ability to separately introduce iron and surfactant, the FeOl-2 precursor allows for a wider and more reliable investigation of the reaction parameter space than does FeOl-1. Two variables are available for tuning nanoparticle size: the OA:Fe ratio and the overall Fe percentage (w/w) in the reaction. The OA in the OA:Fe ratio refers to the amount of OA added, as it assumes no residual/free OA present in FeOl-2 and FeOl-3. Figure 4 demonstrates a complete exploration of the parameter space with size distributions obtained from TEM images (Figure S11). Generally, we find that manipulation of these variables results in two different trends that can be rationalized. As the OA:Fe ratio increases, particle growth is inhibited by the additional surfactant, resulting in smaller particles. As the Fe percentage increases, more metal cluster, i.e., [Fe_3_O(oleate)_6_]^*n*+^, is available for nanoparticle growth, resulting in larger particles. Due to the complex nature and large parameter space of these reactions, these trends are not universal. For example, changes in OA concentration can influence the boiling point of the reaction mixture. The reaction boiling point has been shown to induce nucleation of particles, with higher boiling points resulting in larger nanoparticles.^49^ Thus, as the two parameters (Fe % and OA:Fe) are adjusted, the boiling point is consequently altered, contributing to the size dependence in a way not fully captured by the OA:Fe ratio or the Fe percentage alone. A plot of particle size versus boiling point (Figure S12) exhibits a weak trend, indicating that it is still a contributing factor for particle size control. Finally, the magnetite phase purity was confirmed with powder X-ray diffraction (Figure S13).

**Figure 5:**
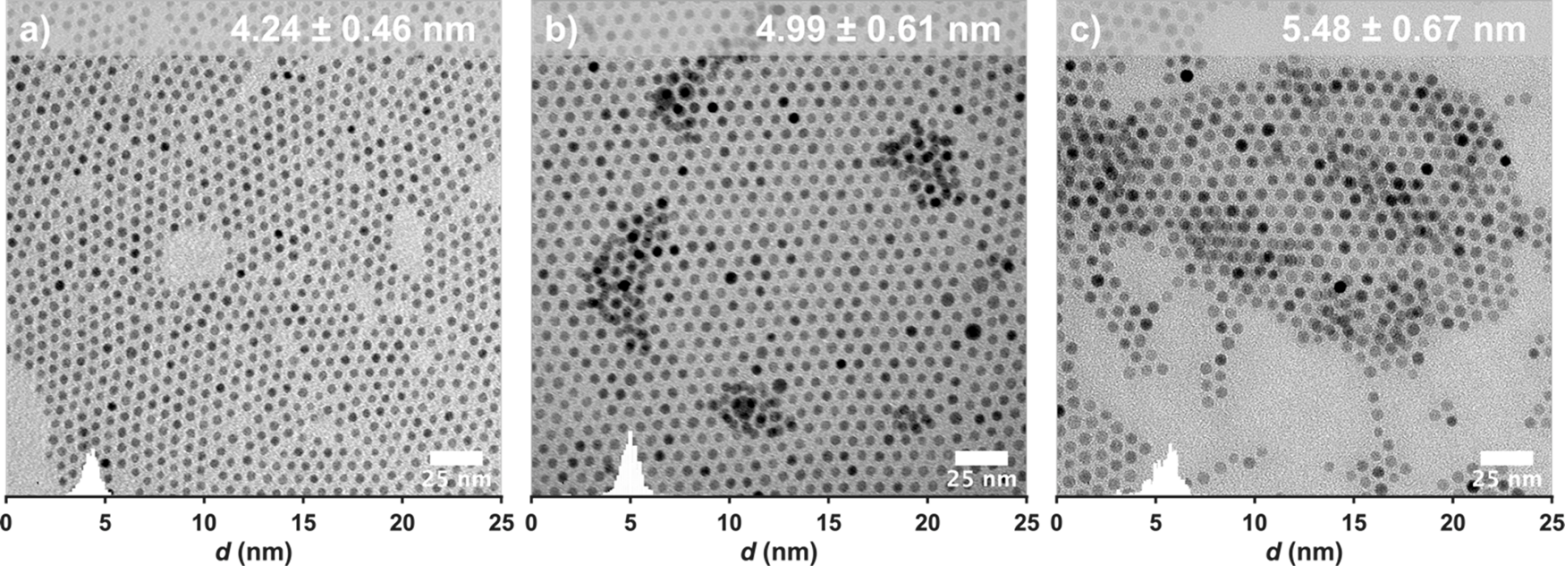
Representative TEM images of nanoparticles synthesized from FeOl-3 with overlaid size histograms.

The synthesis of iron oxide nanoparticles from FeOl-3 as a stock solution in octadecene was performed using a procedure similar to that of FeOl-2. In contrast to the wide size range accessible from FeOl-2, the size range of nanoparticles synthesized from FeOl-3 is limited to 4–5 nm (Figure 5). We attribute this to the structure and composition of FeOl-1. While FeOl-2 is an extended solid that is converted into a reactive molecular cluster with heat and the addition of OA, FeOl-3 is likely far more reactive due to its discrete molecular cluster throughout its entire synthesis. Additionally, FeOl-3 is less sensitive to changes in OA:Fe and Fe %. Thus, the two materials complement each other; FeOl-2 allows for size control in the range of 5–16 nm, while FeOl-3 provides fine size control in the range of 4–5 nm.

The two new starting materials, FeOl-2 and -3, are used in one-pot syntheses that selectively target a specific nanoparticle size without the use of an additional solvent,^18^ seed-mediated growth,^50^ or hot injection.^51^ As the OA:Fe ratio decreases, less OA is available to control the nanoparticle shape, resulting in larger, albeit nonspherical, particles (Figure 3f). Within the scope of the reaction conditions used herein, specifically with ODE as the solvent, particles reach a maximum size of 16.5 nm, with spherical shape control best achieved in the range of 4–12 nm.

In several synthetic procedures, a larger particle size has been achieved by adapting a synthesis to use with a higher-boiling point solvent such as docosane.^42,44,52^ As an initial test of the versatility of our precursors and methods, two reactions were performed with FeOl-2 in docosane, leading to low-size dispersity 13 nm nanoparticles (Figure S14). Additionally, the role of flowing oxygen at reflux was simultaneously studied, with one reaction (Figure S14a) synthesized with 5% O_2_ at reflux and the other (Figure S14b) synthesized with no O_2_ at reflux, demonstrating the importance of O_2_ for the synthesis of phase-pure magnetite.

### Magnetic Characterization of Iron Oxide Nanoparticles

Iron oxide nanoparticles synthesized by thermal decomposition typically consist of magnetite (Fe_3_O_4_), a mixed valence material with a large saturation magnetization and a high magnetic susceptibility.^53^ However, the reducing environment generated by the iron oleate decomposition often leads to inadequate oxidation of Fe^2+^, resulting in wüstite (FeO) core formation inside a magnetite (Fe_3_O_4_) shell. Nanoparticles of FeO@Fe_3_O_4_ exhibit a lower saturation magnetization and lower susceptibilities, limiting the sensitivity in magnetoresistance applications, for example.^54,55^ As previously mentioned, we used a flow of oxygen (5%) during reflux to maintain an oxidizing environment without requiring a postsynthetic oxidation step involving ambient oxygen or a chemical oxidant.^42^

**Figure 6:**
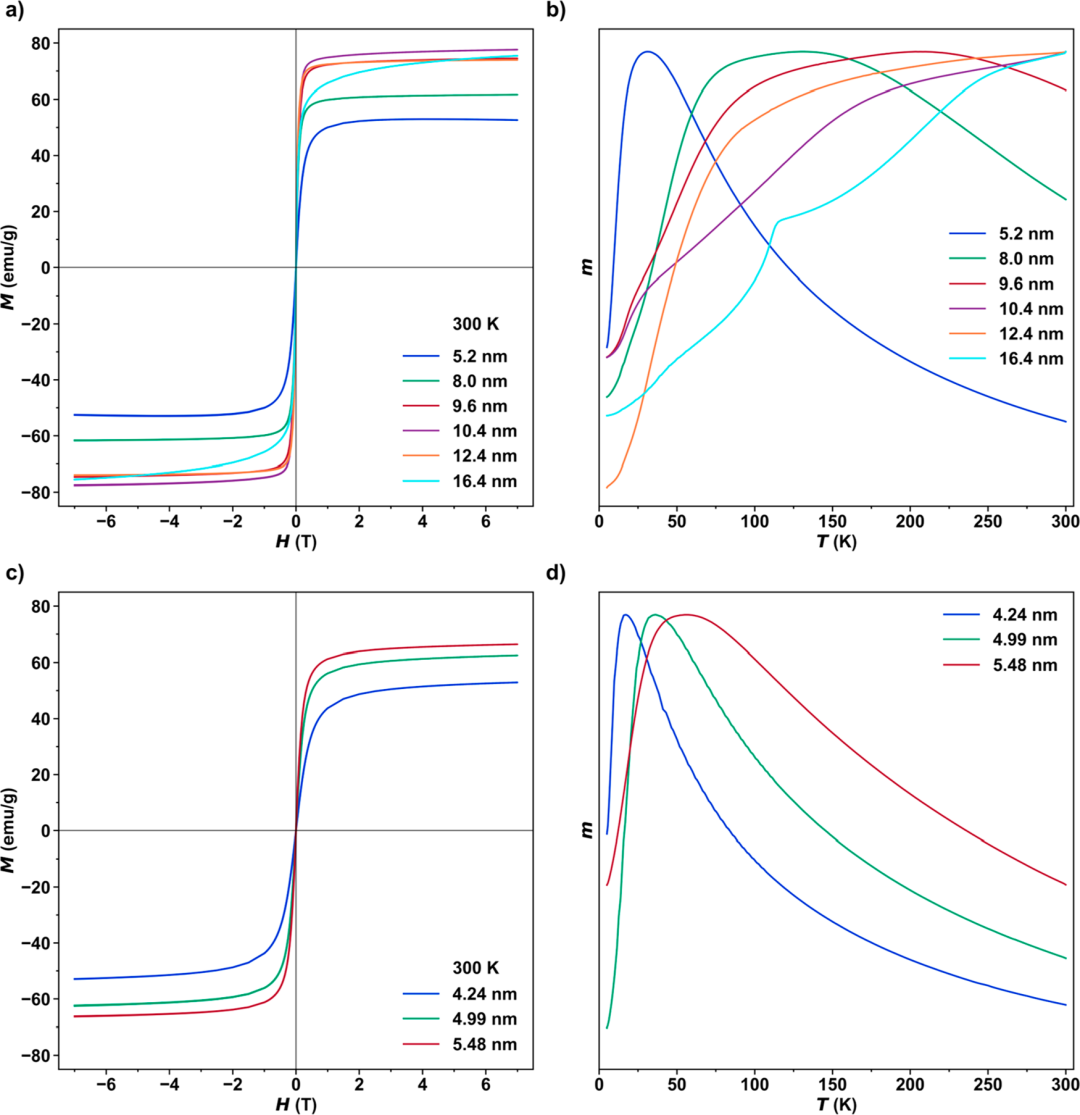
Static magnetic properties of nanoparticles synthesized from FeOl-2 and FeOl-3. Plots of isothermal magnetization vs magnetic field for (a) FeOl-2 and (c) FeOl-3 at 300 K. Plots of normalized zero-field-cooled magnetization vs temperature from 5 to 300 K under an applied field of 0.01 T for (b) FeOl-2 and (d) FeOl-3.

The room-temperature magnetization versus field curves of nanoparticles synthesized from FeOl-2 demonstrate superparamagnetic behavior consistent with magnetite (Figure 6a). As expected, the saturation magnetization generally increases with size. Additionally, a characteristic increase in blocking temperature with size is generally observed in the zero-field-cooled (ZFC) curves (Figure 6b). This behavior is slightly more complex in the larger, faceted particles (10.4 and 12.4 nm), as well as in the largest, nonspherical particles (16.5 nm). Shape effects have been shown to strongly influence the ZFC curves and, thus, the blocking temperature.^17^ Of particular interest from this set of nanoparticle sizes is the emergence of the Verwey transition at ∼105 K for the 16.4 nm particles. The Verwey transition is a metal–insulator transition observed in pure magnetite^56−58^ but is often suppressed due to the presence of defects or nanoscale size and shape effects. The nanoparticles synthesized from FeOl-3 exhibit a similar dependence of size on the saturation magnetization (Figure 6c) and blocking temperature (Figure 6d).

## Conclusions

Two new starting materials for the synthesis of high-quality magnetite have been synthesized, purified, and characterized, namely, a free-flowing powder extended solid form (FeOl-2) and a soluble, waxy solid (FeOl-3). They display several desirable characteristics: a lack of free oleic acid, a consistent synthesis, and long-term stability. Thermal decomposition reactions of FeOl-2 and FeOl-3 yield nanoparticles in tunable size ranges of 5–16 and 4–5 nm, respectively. A subsequent analysis of their static magnetic properties is presented, and trends are consistent with the expected dependencies of saturation magnetization and blocking temperature with size. The consistency of these materials, as well as the method of synthesis, will allow for a more reliable and quantitative mapping of magnetic properties on the nanoscale. Future work will extend the synthetic methods and precursor design ideas herein to enhance the reliability of magnetic properties in nanoparticle syntheses such as transition metal ferrites and antiferromagnetic oxides.

## Supporting Information Available

Experimental details, photographs of experimental setups, data tables, and additional characterization, including pXRD patterns, TEM images, GCMS chromatographs, MALDI-MS spectra, and IR spectra (PDF)
