# Supplementary material for: Size-Tunable Magnetite Nanoparticles from Well-Defined Iron Oleate Precursors
Source: Chem Mater. 2022 Aug 16;34(17):8043–53. doi: 10.1021/acs.chemmater.2c02046 (PMC9477088; doi:10.1021/acs.chemmater.2c02046)
Supplement: Supplementary file 1 — cm2c02046_si_001.pdf [file cm2c02046_si_001.pdf]

Supporting Information

Size-tunable magnetite nanoparticles from well-defined iron oleate precursors

Kyle M. Kirkpatrick[†], *Benjamin H. Zhou*[‡], *Philip C. Bunting*[†], *and Jeffrey D. Rinehart*[*,†,‡]

[†]Department of Chemistry and Biochemistry and [‡]Materials Science and Engineering Program, University of California – San Diego, La Jolla, California 92093, United States

Table S1. Batch to batch composition of FeOl-2 from EA and ICP-MS.

FeOl-2	C	H	Fe
Batch A	60.32	8.28	15.45
Batch B	60.18	8.74	14.97
Batch C	60.76	8.56	15.39
Average	60.42 ± 0.30	8.50 ± 0.14	15.29 ± 0.26

Table S2. Summary of nanoparticle synthesis parameters

Nanoparticles synthesized from FeOl-2						
**FeOl-2 (g)**	**OA (g)**	**ODE (g)**	**OA:Fe (mol)**	**Fe % (w/w)**	**Size (nm)**	**Stdev**
0.25	0.10	7.30	0.5	0.5	10.55	1.52
0.25	0.19	7.20	1.0	0.5	8.02	0.86
0.25	0.29	7.13	1.5	0.5	7.75	1.35
0.25	0.38	7.00	2.0	0.5	5.19	0.58
0.50	0.20	6.95	0.5	1.0	12.48	1.77
0.50	0.39	6.80	1.0	1.0	12.41	1.69
0.50	0.59	6.59	1.5	1.0	10.42	1.22
0.50	0.78	6.40	2.0	1.0	8.64	0.87
0.75	0.29	6.61	0.5	1.5	16.41	2.63
0.75	0.57	6.30	1.0	1.5	8.19	1.03
0.75	0.87	6.02	1.5	1.5	8.15	1.70
0.75	1.16	5.74	2.0	1.5	12.30	1.97
1.00	0.46	6.27	0.6	2.0	16.65	2.91
1.00	0.77	5.87	1.0	2.0	9.62	1.01
1.00	1.17	5.48	1.5	2.0	12.81	1.74
1.00	1.55	5.09	2.0	2.0	8.80	2.03

Nanoparticles synthesized from FeOl-3						
**FeOl-3 (g)**	**OA (g)**	**ODE (g)**	**OA:Fe (mol)**	**% Fe (w/w)**	**Size (nm)**	**Stdev**
0.87	0.59	7.00	1.5	0.9	4.24	0.46
1.26	0.40	7.00	0.7	1.3	4.99	0.61
1.50	1.01	7.00	1.5	1.4	5.48	0.67

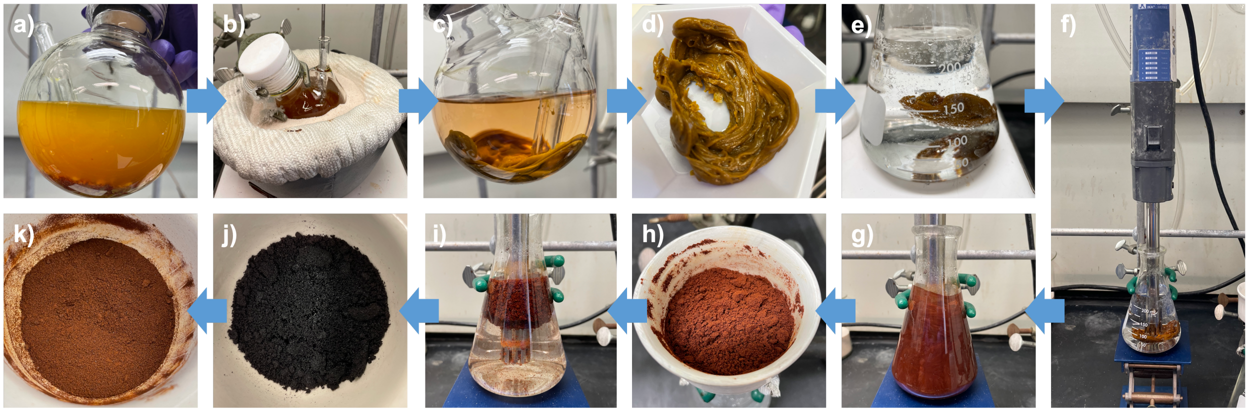

Figure S1. Step by step synthesis of FeOl-2. (a) Combined reactants in pressure flask. (b) Setup of flask in heating mantle. (c) Reaction end. (d) Rubbery solid resulting from the reaction. (e) Solid in ~ 250 mL DI water in 250 mL Erlenmeyer flask. (f) Tissue homogenizer inserted into flask. (g) Homogenization. (h) Product recovered after vacuum filtration. (i) Second homogenization (product floats to surface when not running homogenizer) (j) Dried 24 h in vacuum oven, then crushed with pestle. (k) Final product, FeOl-2, after additional 24 h in vacuum oven.

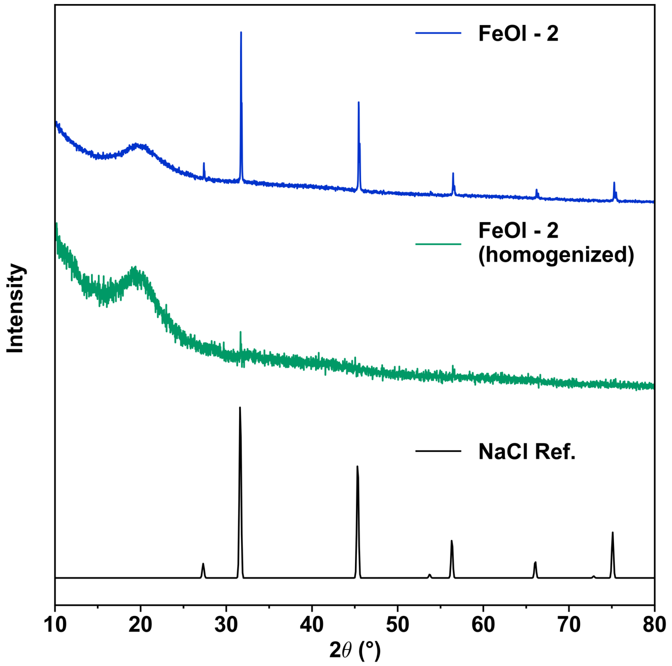

Figure S2. Powder x-ray diffraction patterns of non-homogenized and homogenized FeOl-2 with NaCl reference pattern, demonstrating the utility of a tissue homogenizer in removing NaCl.

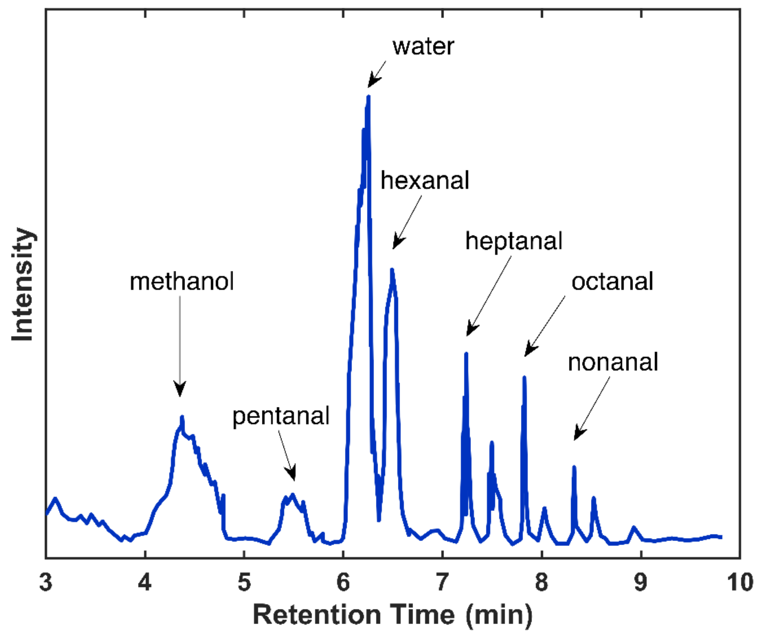

Figure S3. GCMS chromatograph of the headspace during the reaction of FeOl-2 and oleic acid at 70 °C. Peaks without corresponding labels were unable to be identified by the MS software.

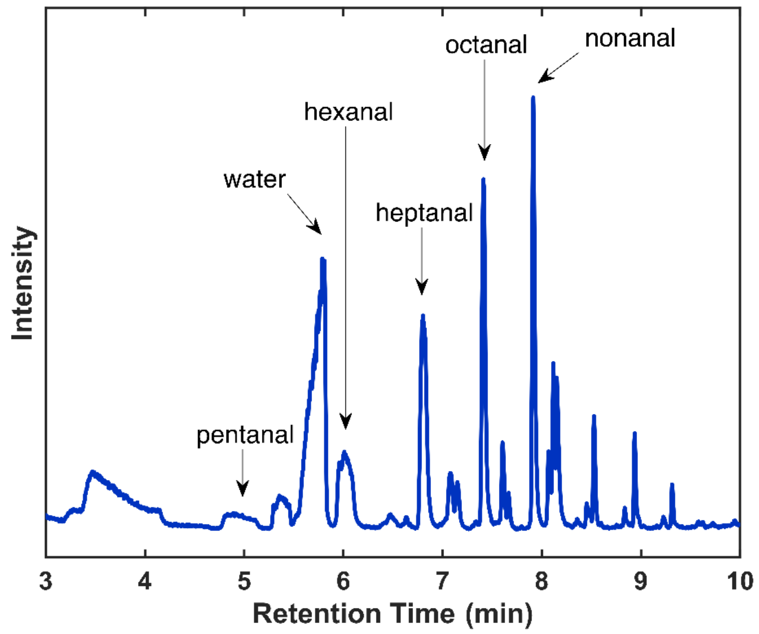

Figure S4. GCMS chromatograph of the headspace during the reaction of FeOl-1 and oleic acid at 70 °C. Peaks without corresponding labels were unable to be identified by the MS software.

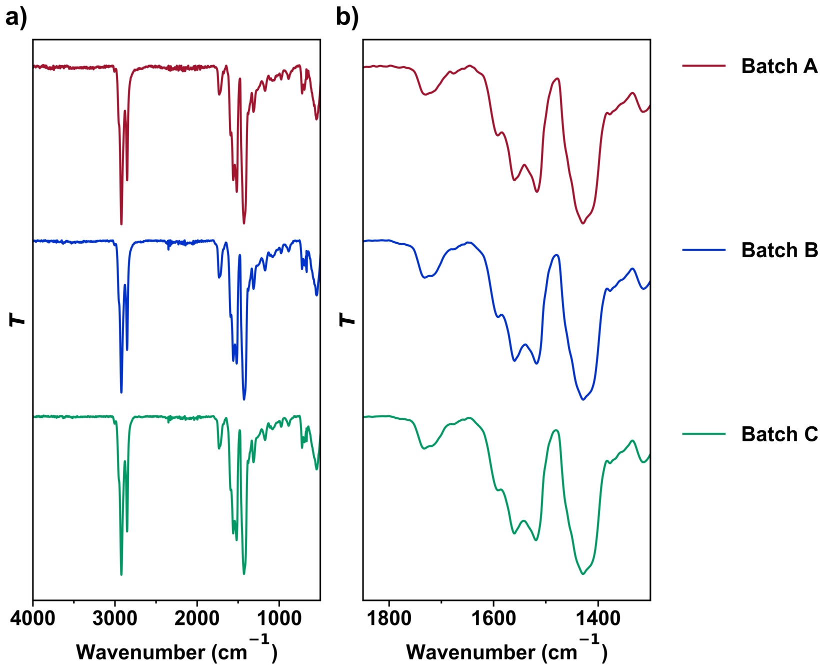

Figure S5. Full IR spectra for three batches of FeOl-2 prepared in identical fashion. (a) Full spectrum and (b) metal carboxylate region.

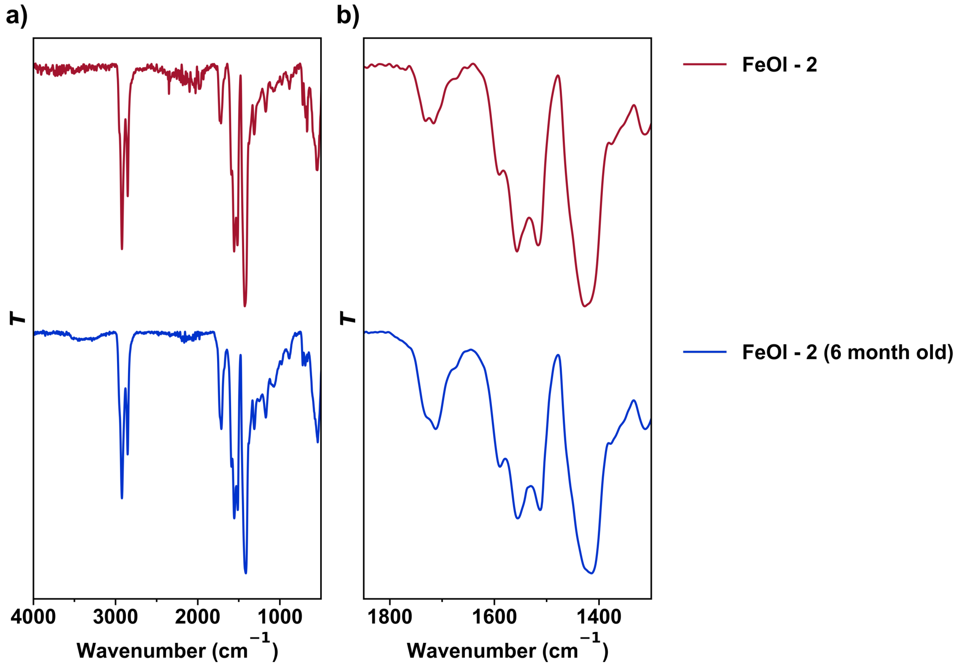

Figure S6. Full IR spectra (a) and metal carboxylate region (b) of freshly synthesized FeOl-2 vs. six-month-old FeOl-2 stored under ambient conditions.

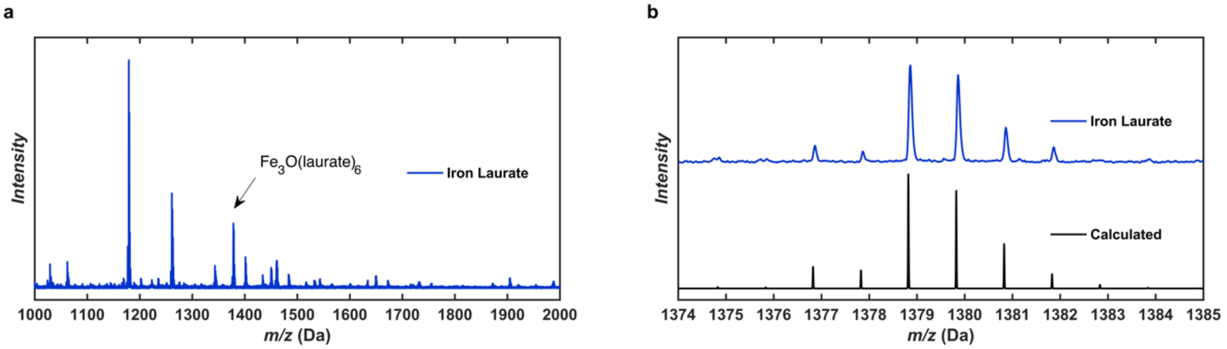

Figure S7. MALDI-MS data demonstrating ligand replacement on the central metal-oxo cluster. (a) Full MALDI-MS spectra of $Fe_3O(laurate)_6$, (b) and molecular ion with calculated isotope pattern.

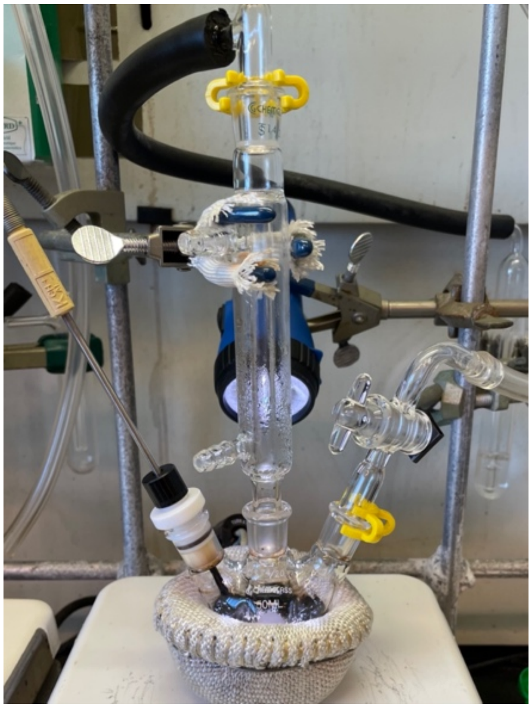

Figure S8. Experimental setup for the synthesis of magnetite nanoparticles.

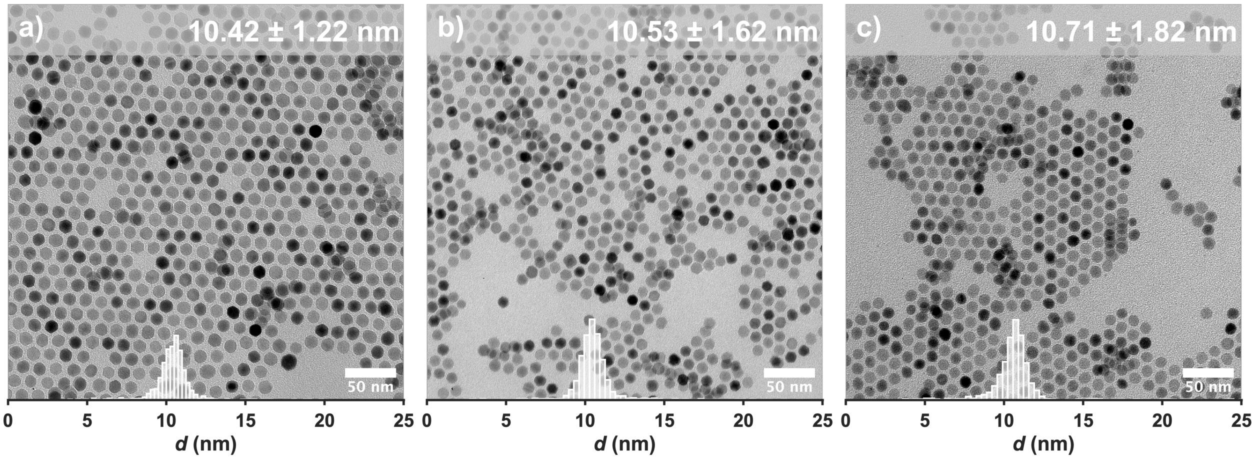

Figure S9. TEM images of repeated reactions for the synthesis of 10.5 nm nanoparticles from FeOl-2.

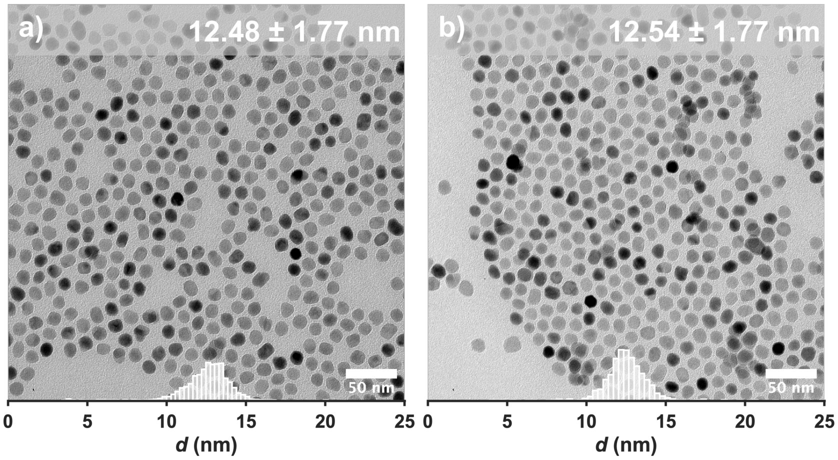

Figure S10. TEM images of repeated reactions for the synthesis of 12.5 nm nanoparticles from FeOl-2.

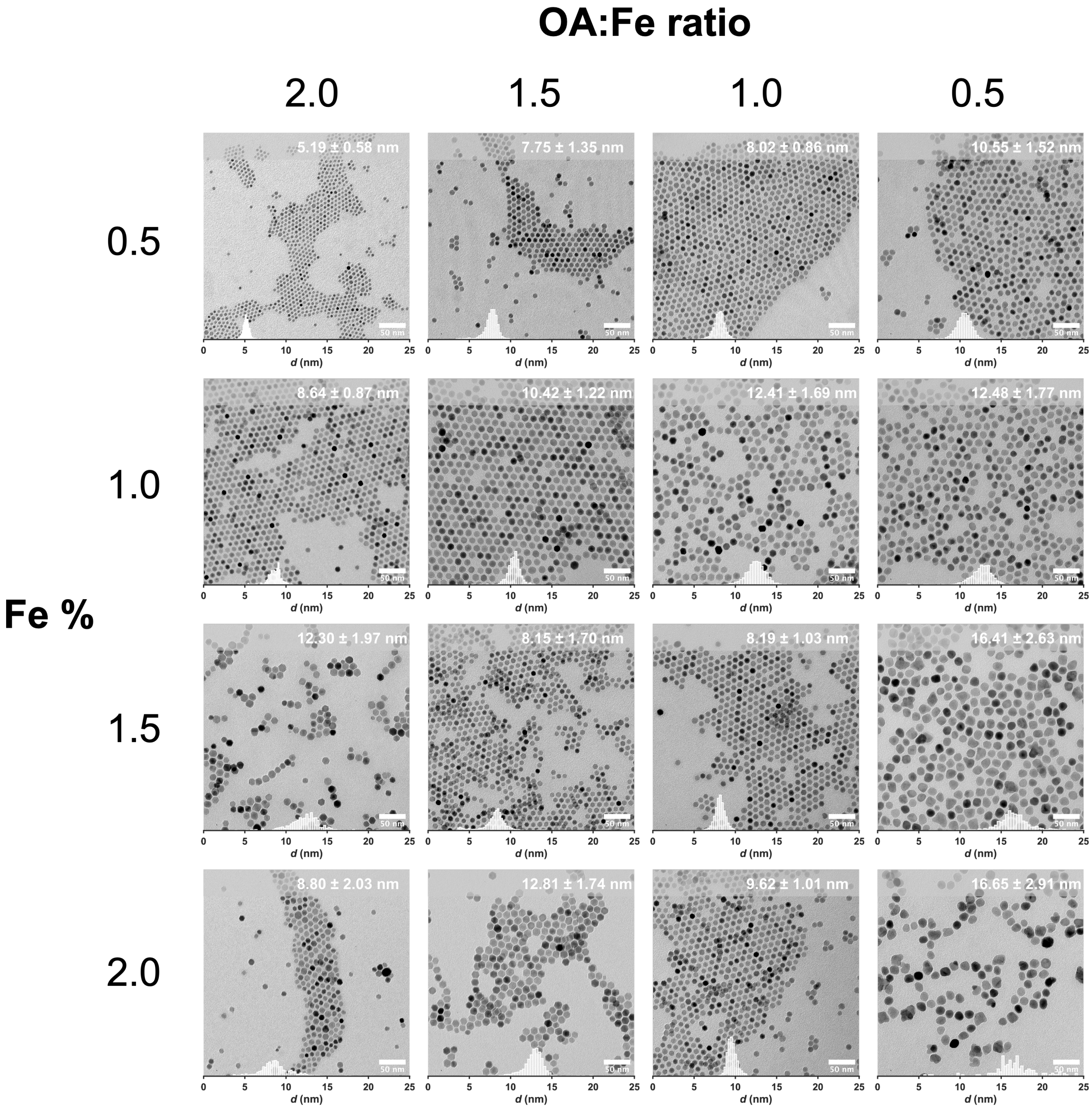

Figure S11. TEM images from full exploration of the parameter space, varying the OA:Fe molar ratio (top) and Fe % (bottom). Synthetic parameters for each synthesis are in Table S2.

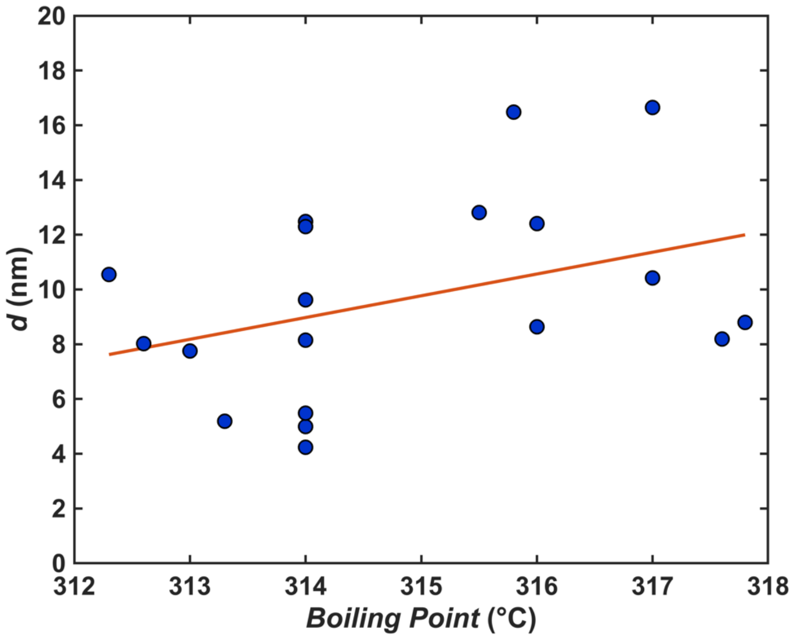

Figure S12. Plot of particle diameter (nm) vs. boiling point (°C) of the nanoparticle reaction showing weak correlation (y = 0.7951x – 240.67 $R^2 = 0.1425$).

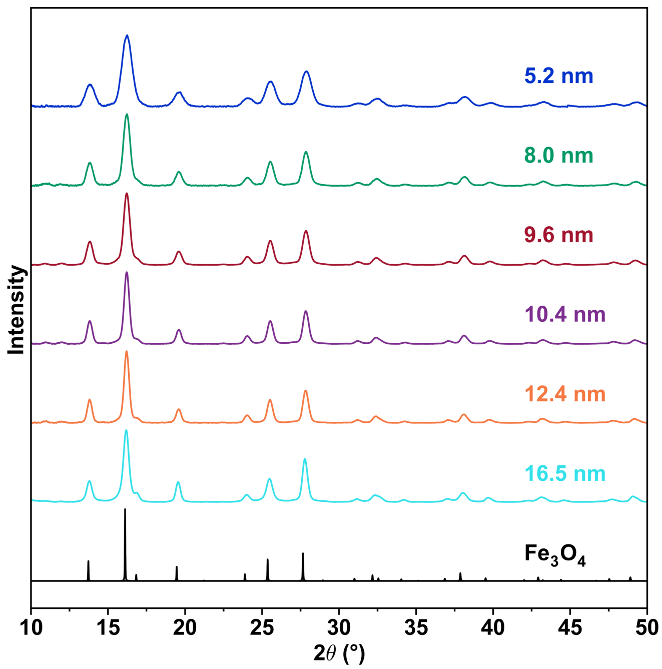

Figure S13. Powder x-ray diffraction (PXRD) patterns for the six representative magnetite nanoparticle sizes shown in Figure 3, collected using a Mo Kα (λ = 0.71073 Å) source.

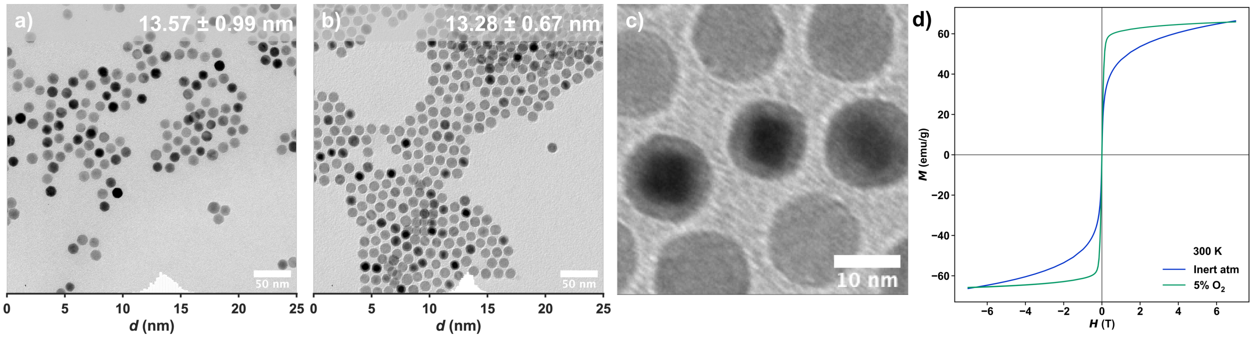

Figure S14. TEM images of nanoparticles synthesized from FeOl-2 in docosane. (a) Phase-pure nanoparticles from 5% O_2 flow at reflux. (b) Core-shelled nanoparticles from no O_2 flow at reflux. (c) Observed core-shelling. (d) Plot of isothermal magnetization vs. magnetic field for phase-pure and core-shelled nanoparticles at 300 K.
